# Psychosocial interventions for people with comorbid substance use disorders in people with severe mental health conditions in low- and middle-income countries: scoping review

**DOI:** 10.1192/bjo.2025.10840

**Published:** 2025-09-22

**Authors:** Awoke Mihretu, Abebaw Fekadu, Azeb Asaminew Alemu, Charlotte Hanlon

**Affiliations:** Department of Psychiatry, WHO Collaborating Centre for Mental Health Research and Capacity Building, College of Health Sciences, Addis Ababa University, Addis Ababa, Ethiopia; Centre for Innovative Drug Development and Therapeutic Trials for Africa (CDT-Africa), Addis Ababa University, Addis Ababa, Ethiopia; Department of Global Health & Infection, Brighton and Sussex Medical School, Brighton, UK; Division of Psychiatry, Centre for Clinical Brain Sciences, University of Edinburgh, Edinburgh, Scotland, UK

**Keywords:** Psychosocial interventions, comorbid substance use disorders, severe mental health conditions, dual diagnosis, recovery

## Abstract

**Background:**

The high comorbidity of substance use disorders (SUDs) among people with severe mental health conditions (MHCs) poses major challenges to providing effective care, particularly in low- and middle-income countries (LMICs), where treatment options are limited.

**Aims:**

The aim of this scoping review was to produce an overview of the current evidence on psychosocial interventions for people with comorbid MHCs and SUDs in LMICs.

**Method:**

The following databases were searched from their inception to 23 July 2024: PubMed/Medline, Global Health, Embase, PsycINFO and Global Index Medicus. We also searched for grey literature, using Google Scholar, ProQuest and Clinicaltrials.gov. Reporting was according to the Preferred Reporting Items for Systematic Reviews and Meta-Analyses extension for Scoping Reviews (PRISMA-ScR) Checklist. Studies were eligible if they focused on any psychosocial intervention targeting substance use in people with severe MHCs from LMICs. Of the 6304 records screened by titles and abstracts, 138 full-text articles were assessed and included for data extraction.

**Results:**

Of the 6304 records screened by titles and abstracts, 138 full-text articles were assessed and 13 articles were included for data extraction. Many of the studies (*n* = 9) had a quasi-experimental design, and were from Latin America and South Asia. Four studies were randomised controlled trials. The primary outcomes examined were substance use abstinence, treatment engagement and retention, reduction in psychiatric symptoms, functioning and suicidal behaviours. Despite some heterogeneity in study designs, target populations and evaluated outcomes, interventions including various tobacco cessation programmes, screening and brief intervention with family support, and community-based programmes, have demonstrated positive outcomes in reducing tobacco, alcohol and khat use, respectively.

**Conclusions:**

The review shows that there have been few initiatives to design and test psychosocial interventions for individuals with comorbid severe MHCs and SUDs in LMICs. There is a clear need to design and test feasible, acceptable, and effective interventions to address both severe MHCs and substance use when they co-occur.

Substance use disorder (SUD) – encompassing all forms of problematic substance use, including substance misuse, dependence, harmful use and hazardous use – is the most common and clinically significant comorbidity among individuals with severe mental health conditions (MHCs). Severe MHCs refer to major psychiatric disorders such as schizophrenia, bipolar disorder and major depressive disorder, particularly when these conditions are associated with prolonged disability. The term ‘dual diagnosis’ or ‘dual disorders’ is used to describe the co-occurrence of a severe MHC and an SUD.^1,2^

In low- and middle-income countries (LMICs), mental and SUDs are among the leading contributors to the overall disease burden, accounting for an estimated 5.4% of the total burden. These conditions have substantial social and economic consequences.^3,4^ Mental illness and SUDs comprise an estimated 7.4% of global disability-adjusted life-years (DALYs) and 22.7% of global years lived with disability (YLDs).^5^ DALYs represent the total burden of disease and are calculated as the sum of years of life lost (YLLs) and YLDs. YLLs are estimated by multiplying the number of deaths by the standard life expectancy at the age of death. YLDs are calculated by multiplying the prevalence of a health condition by its assigned disability weight, adjusted for age, gender, location and year.^6^ The prevalence of SUDs among individuals with severe MHCs is markedly higher than in those without severe MHCs, posing significant challenges in clinical practice. Estimates from meta-analyses indicate that comorbid alcohol and/or other SUDs are observed in approximately 25% of individuals with major depressive disorder^7^ and 42% of individuals with schizophrenia.^8^ Among people with bipolar disorder, the prevalence of alcohol use, cannabis use and illicit drug use has been estimated to be 42, 20 and 17%, respectively.^9^

In LMIC settings, the prevalence of co-occurring problematic SUDs and severe MHCs is reported to be high, ranging from 50 to 68%, primarily based on studies conducted in clinical populations.^10,11^ In Ethiopia, where this review is situated, comorbid SUD is estimated to affect approximately a third of individuals with severe MHCs, with khat being the most commonly reported substance, followed by alcohol and tobacco.^12–15^ The prevalence of any substance use among people with severe MHCs in Ethiopia has been reported to range from 25 to 40%,^13–16^ and the prevalence of alcohol use disorder specifically ranges between 10 and 25%.^12,15^

A minimal level of problematic substance use or any substance use among individuals with severe MHCs can have far-reaching negative effects, encompassing exacerbated psychiatric symptoms, recurrent hospital admissions, treatment non-adherence, compromised personal recovery, suicidal ideation or attempts, incarceration, homelessness, physical health complications, mortality^16–20^ and physical health comorbidities.^21^

To address common risk factors or mitigate the adverse impact of problematic substance use in individuals with severe MHCs, various psychosocial interventions have been tested, but many studies were from high-income countries (HICs).^22^ These interventions are rarely tested or adapted for LMICs.^22^

Recommendations and guidelines from HIC organisations such as the Substance Abuse and Mental Health Services Administration (SAMHSA), National Institute of Health (NIH) and American Psychological Association (APA)^23^ have limited applicability to low-resource contexts. For example, assertive community treatment and token economy interventions, practiced in HICs, require significant human and material resources that may not be feasible in LMICs. There may also be limited peer support, insufficient availability of opioid antagonist treatments and a lack of robust frameworks for the early detection of problematic substance use among individuals with severe MHCs.^22^

As we anticipated that there would be very few primary studies on the topic in LMICs, and previous systematic reviews or meta-analyses have focused on single psychosocial interventions or specific substances, such as psychoactive drugs or alcohol, predominantly from HIC settings, we undertook a scoping review. We aimed to identify existing evidence on a broad range of psychosocial interventions for comorbid SUDs of all types in people with severe MHCs in LMICs, including any type of intervention study design and extending to include the grey literature.

## Method

This scoping review protocol was registered at Open Science Framework (OSF): https://osf.io/867rf. We conducted and reported this scoping review according to the Preferred Reporting Items for Systematic Reviews and Meta-Analyses Extension for Scoping Reviews (PRISMA-ScR) Checklist.^24^

### Eligibility criteria

For this scoping review, articles, student theses and other grey literature were included. According to the PICOS framework for intervention studies (Population, Intervention, Control, Outcome and Setting), eligible studies included participants of all ages and psychosocial interventions targeting SUDs in people with severe MHCs. Psychosocial interventions encompassed psychological approaches aimed at enhancing motivation for behavioural change, medication adherence and treatment engagement, symptom reduction and relapse prevention. Psychosocial interventions also included interventions that focused on improving functioning and quality of life, and developing social skills, assertiveness and personal growth. Eligible studies also included any study relating to interventions for this group, including randomised controlled trials, controlled before-after, pre-post, pilot, naturalistic and other intervention evaluation studies of people with comorbidity, as well as qualitative and mixed-methods studies that described interventions, their implementation and experiences of the intervention. Studies were required to have any comparison condition, including treatment as usual, a comparison between two interventions, waiting list controls and comparing pre- and post-intervention outcomes; and be conducted in LMICs, as per the World Bank classification at the time the study was conducted or reported. The specific settings could include community settings, prisons, primary or specialised healthcare settings, residential settings, traditional or religious healing sites, or any other community setting. The review focused on substance use only when it qualified as a form of problematic use – such as dependence, harmful use, hazardous use or a diagnosed SUD. The assessment tools included were those specifically designed to identify problematic substance use. This criterion was also applied to tobacco use, with a particular focus on nicotine dependence. Severe MHCs encompassed all forms of psychotic disorders, mood disorders and depressive disorders, as diagnosed by trained mental health professionals.

### Exclusion

We excluded non-research publications (e.g. commentaries, editorials). Review papers were examined for the primary studies they included, and these studies were evaluated against the eligibility criteria to determine inclusion or exclusion.

### Information sources

We searched five databases: PubMed/Medline, Global Health, Embase, PsycINFO and Global Index Medicus. For grey literature, including Master’s or PhD theses, we searched ProQuest. We also searched Clinicaltrials.gov for protocols of intervention study and conducted a targeted search in Google Scholar. We searched from database inception to 29 December 2023, and updated the review on 23 July 2024. We conducted both backward and forward citation searches of all included studies.

### Search criteria

Our search strategy consisted of key terms, free text and controlled vocabulary search terms such as Medical Subject Heading/MeSH terms for PubMed/Medline, PsycINFO terms and Emtree terms for Embase (see Supplementary File 1 available at https://doi.org/10.1192/bjo.2025.10840). The main concepts connected by the Boolean operator ‘AND’ were severe MHCs, SUDs, dual diagnosis, psychosocial interventions and LMICs.

### Selection of sources of evidence

After searching the databases, the results were exported to Rayyan, a web-based tool, used on Windows (Rayyan Systems Inc., Doha, Qatar; https://www.rayyan.ai).^25^ Following de-duplication, two independent reviewers, A.M. and A.A.A., performed title and abstract screening. A.M. and A.A.A. also conducted full-text screening and extracted data from included articles. Conflicts were resolved by senior authors of the study.

### Data charting process

The data extraction form included the following: study name or title; year of publication; country/countries; setting details; study design; population and participant details; type, dose, delivery agent, model and other characteristics of the psychosocial intervention (psychotherapy, system-based intervention, social intervention, family intervention, psychoeducation); type of substance use; type of MHCs; assessment time points; primary and secondary outcome measures; outcomes and impacts.

### Collating, summarising and reporting the results

We summarised the data with descriptive counts and frequencies, and presented the key findings in a narrative format.

## Results

**Fig. 1:**
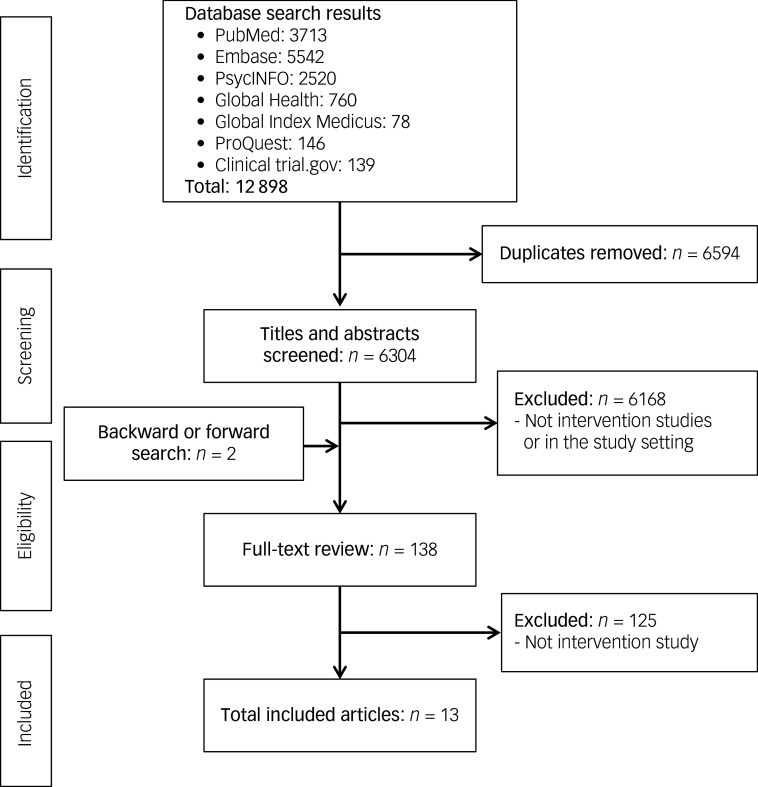
PRISMA flow diagram of the study selection process.

After de-duplication, 6304 records were identified and title/abstract screening undertaken. Of these, 138 full-text articles were assessed for eligibility, and 13 articles met the inclusion criteria for the review (Fig. 1). The majority (76%) of the assessed studies were not intervention studies. The remaining articles were excluded because they had the wrong population (i.e. did not address comorbid SUD and severe MHCs) or were conducted in settings outside LMICs.

### Overall characteristics of the studies

The studies were conducted across diverse settings, but from a small number of LMICs (Table 1): four studies from Brazil, two each from Iran and Thailand, and one study each from Ethiopia, Kenya, Somalia, India and Mexico. The settings included various centres specialising in psychosocial intervention for alcohol and other drug rehabilitation in Brazil,^26^ community-based residential care facilities in Mexico,^27^ therapeutic community centres in Iran,^28^ hospital discharges in the community in Somalia,^29^ drop-in centres in Iran,^30^ psychiatric out-patient units within tertiary care hospitals in India,^31^ primary healthcare-based patients in the community in Ethiopia^32^ and psychiatric in-patients in Thailand.^33^ Moreover, the study engaged special populations, including Somali refugees in Kenya.^34^ Participants across the studies were individuals with psychosis, psychotic depression and comorbid SUDs. The MHCs examined in the included studies were schizophrenia alone (*n* = 1), severe psychiatric or MHCs (*n* = 4), psychosis (*n* = 4), depressive disorders (*n* = 3) and mood disorders (*n* = 1).The substances examined in the studies included tobacco (*n* = 5), opioids (*n* = 1), alcohol alone and alcohol with other drugs (*n* = 3), SUDs (*n* = 2) and khat (*n* = 2).

**Table 1:** Characteristics and results of the included studies

Author, year (country)	Setting	Study design	Participants	Type of intervention and session duration	Delivery agents	Sample size	Outcome measure	Results
Odenwald et al, 2012 (Somalia)^29^	Community and hospital discharges	Pre-post pilot	Patients with psychosis and khat misuse and their carers	An intensive 6-week home-based treatment package (psychoeducation, monthly home visits and counselling)	Trained staff of our local partner non-governmental organisation	35 consecutively	BPRS, functioning, khat use and other outcomes	Mixed findings Most symptoms worsen Khat use could only be reduced in the group of out-patients
Castaldelli-Maia et al, 2013 (Brazil)^36^	Community psychosocial units	Pre-test/post-test only, no comparison group	Psychiatric disorders and addictions	Six scheduled sessions of group therapy and four scheduled visits with a psychiatrist for 6 weeks	Six scheduled sessions of group therapy and four scheduled visits with a psychiatrist (t1 = 0; t2 = 1 week; t3 = 3 weeks; t4 = 6 weeks)	90 (15 people for each 6-week treatment)	Success as defined as abstinence from smoking for a period of at least 14 consecutive days	Participants with hypertension and alcohol use showed success rates (34.2–44.4%) Good success rates were observed for pharmacological treatment, which was combined with group therapy Integrating smoking cessation programmes into ongoing treatment of other addictions and psychiatric disorders are helpful and recommended Smoking one’s first cigarette for 5 min or more after waking was a predictor
Castaldelli-Maia et al, 2014 (Brazil)^35^	Community psychosocial units	Pre-test/post-test only, no comparison group	Clinical psychiatric disorders and nicotine dependence	Six sessions of group therapy, and four medical consultations with a psychiatrist	Psychiatry residents under fully qualified psychiatrists, medical doctors and psychologists	367	The presence of the individual in all four medical consultations and six group sessions (retention)	Nicotine replacement therapy seems to be very important in the beginning of the treatment Time to smoking the first cigarette 5 min or later after waking and nicotine patch use were associated with retention
Thapinta et al, 2014 (Thailand)^33^	Hospital patients	Pre-test/post-test	Alcohol dependence and depression	Three weeks of brief six-session CBT (40–60 per session)	Master’s degrees in mental health and psychiatric nursing	60	Eight-question Suicidal Risk Scale and nine-item depression scale	Promising results may benefit patients with alcohol dependence and depression
Tantirangsee et al, 2015 (Thailand)^37^	Hospital patients	RCT	Problematic substance uses and schizophrenia/ psychosis	Single-session brief intervention 30–45 mins, or a single session and brief intervention with family support in attendance	Psychiatric nurses	169	ASSIST, Global Assessment of Relational Functioning	The brief intervention with family support group had better relational functioning in comparison to those receiving brief intervention only A brief intervention reduced smoking involvement
Zemestani and Ottaviani et al, 2016 (Iran)^28^	Therapeutic community centre	Quasi-experimental; pre-test/post test	Individuals with SUD and depression disorders	8 weeks for 2 h out-patient MBRP programme compared with treatment as usual	Master’s degree in psychology or social work	74 participants	Beck Depression Inventory-II, Penn Alcohol Craving Scale and SCID-I	Lower post-intervention rates of depression and craving in those who received MBRP as compared with those in treatment as usual
Castaldelli-Maia et al, 2017 (Brazil)^38^	Psychosocial care units in the community	Qualitative report of the training and implementation process	Psychiatric disorders and addictions	8-h training package for the staff of psychosocial care staffs	A medical doctor, psychiatrist and a psychologist	186 health professionals	Topics focused on the implementation of treatment for mental health and addiction smokers: management, epidemiology, medications, psychotherapy and smoking/mental health assessment instruments	Resistance to incorporating tobacco dependence treatment Resistance to the implementation of CBT and treatment for smoking is already implemented in primary care network; resistance to the use of medication in addiction treatment
Widmann et al, 2017 (Kenya)^34^	Refugee camp and, in the community	RCT	Khat users and psychopathology including psychosis	Screening and brief intervention, short psychotherapy group	College graduates from the local community	330 Somali khat users, all male, of whom 23% (*n* = 73) reported experiencing khat-related psychosis symptoms	ASSIST, PHQ-9, a Somali short version of the PDS and parts from the CIDI	Statistically significant increase in functioning hours from 8.55 h to 11.68 h Statistically significant reduction in hours of khat chewing from 7.61 to 4.64
Lima et al, 2020 (Brazil)^26^	Community mental healthcare	Uncontrolled before-after	SUD only, severe MHCs only, and SUD plus severe MHCs	Six scheduled sessions of tobacco cessation group therapy Scheduled visits with a psychiatrist	Physicians (psychiatry residents) and medical specialists or psychiatrists, when necessary	498	Self-report 4-week quitter Treatment retention	29.1% were abstinent from tobacco for ≥4 weeks 42% completed the programme Individuals with SUD plus severe MHCs had worse response to treatment
Marín-Navarrete et al, 2021 (Mexico)^27^	Community-based residential care facilities	Two-stage implementation study, no control group	Psychosis, mania (MINI) and cognitive impairment (MoCA) positive patients, and comorbid alcohol and drug use	Dual diagnosis screening interview using MINI as a gold standard	Addiction and mental health professionals	A convenience sample of 213	Dual diagnosis screening	The dual diagnosis screening obtained sensitivity and specificity scores above 85% The mobile application-based dual diagnosis screening was acceptable
Alavi et al, 2023 (Iran)^30^	Drop-in centre	RCT	Opioid use disorder, depression and psychosis	Four sessions of brief CBT with 90 min each	Trained psychologist	40 purposively selected and randomly assigned	GHQ-28 followed by a psychiatrist diagnosis	No significant statistical difference in all subclasses and GHQ-28 for both groups No statistically significant difference in terms of psychosis (*P* = 0.077)
Sinha et al, 2022 (India)^31^	Psychiatric out-patient setting of a tertiary care multi-specialty hospital	A parallel-group, single-blind, randomised controlled trial	Hazardous alcohol use and mood disorders	Individual-based, face-to-face screening, and brief intervention took 15–20 min	A graduate doctor, pursuing 3 years of training in psychiatry	84 with random selection and assignment	AUDIT-9, Hamilton Rating Scale for Depression and the Young Mania Rating Scale	Screening and brief intervention reduces alcohol risk scores Intervention improves motivation to reduce risky alcohol use
Asher et al, 2023 (Ethiopia)^32^	Community	RCT	People with alcohol use disorder and schizophrenia	Community-based rehabilitation (5 weeks of initial training)	Community-based rehabilitation workers who were lay persons recruited from the local area	20% from 160 with comorbid alcohol use disorder	AUDIT scores ≥ 8	No evidence of community-based rehabilitation effect on alcohol use disorder at 12 months

BPRS, Brief Psychiatric Rating Scale; CBT, cognitive–behavioural therapy; RCT, randomised clinical trials; ASSIST, Alcohol, Smoking and Substance Involvement Screening Test; SUD, substance use disorder; MBRP, mindfulness-based relapse prevention; SCID-I, Structured Clinical Interview for DSM-IV Axis I Disorders; PHQ-9, Patient Health Questionnaire-9; PDS, Post-traumatic Diagnostic Scale; CIDI, Composite International Diagnostic Interview; MHC, mental health conditions; MINI, Mini-International Neuropsychiatric Interview; MoCA, Montreal Cognitive Assessment; GHQ-28, General Health Questionnaire-28; AUDIT, Alcohol Use Disorders Identification Test.

Only four of the 13 studies were randomised controlled trials. Participant numbers in the studies ranged from 35 to 498, with six studies having a sample size below 100. Eight studies were conducted in community settings, two took place in general hospitals and one in a specialised hospital. In eight studies, the intervention was delivered by mental health professionals, including psychiatrists, psychologists or psychiatric nurses. Intervention providers without formal degrees participated in three studies, particularly those focused on psychosocial rehabilitation.

### Primary outcomes of the studies

The primary outcomes of the studies included substance use abstinence, treatment engagement and retention, psychiatric symptoms, functioning and suicidal behaviour. Specifically, these outcomes encompassed smoking cessation or tobacco abstinence and treatment retention,^26,36^ reduction in psychotic symptoms measured by the Brief Psychiatric Rating Scale (BPRS), improvement in functioning and a decrease in hours spent chewing khat.^28^ Additional outcomes assessed were depressive symptoms, evaluated with the Beck Depression Inventory-II (BDI-II) or the Structured Clinical Interview for DSM-IV Axis I Disorders (SCID-I),^28^ and khat use quantified by the Alcohol, Smoking and Substance Involvement Screening Test (ASSIST).^32^

### Interventions

The studies focused on implementation of dual diagnosis screening (*n* = 1), tobacco cessation programmes (*n* = 4), psychological interventions (*n* = 3) and psychosocial rehabilitation (*n* = 3). None of the studies employed a rigorous cultural adaptation process. One study found that a dual diagnosis screening tool, delivered through a mobile phone application, was easy to apply, acceptable and had demonstrated sensitivity and specificity scores above 85%.^27^ There were four studies focusing on tobacco cessation among people with severe MHCs.^26,35,36,38^ The success rate for tobacco abstinence ranged from 29.1 to 44.4%. In one study, the attendance rate for tobacco cessation programmes was 42%. Factors associated with successful completion of these programmes included pre-intervention time to first cigarette of 5 min or more after waking and the use of nicotine patches as part of nicotine replacement therapy.^36^ However, there was also resistance in integrating tobacco dependence treatment within primary care networks, particularly regarding the adoption of cognitive–behavioural therapy (CBT) and medication use in addiction treatment.^38^ In studies investigating tobacco interventions for individuals with severe MHCs, findings emphasised the importance of tailoring treatment methods based on the specific combination of comorbidities and the individual’s motivation stage. Additionally, the role of psychosocial techniques in increasing motivation and resolving environmental and situational challenges was highlighted. Effective psychiatric support to manage psychotic symptoms, mania and depression (with or without suicide risk) could also contribute to better engagement in substance use interventions.^39^

Psychological interventions, such as CBT, group therapy, screening and brief intervention, brief intervention with family support and mindfulness-based relapse prevention (MBRP), have been utilised. Both screening and brief intervention and MBRP have been shown to enhance motivation and reduce alcohol risk scores. There was no statistically significant difference in psychosis symptoms post-intervention in the MBRP group (*P* = 0.077).^30^ However, participants receiving MBRP reported significantly greater improvements in depression and cravings compared with those on treatment as usual.^28^ Six-session (40–60 min per session) CBT was also promising in benefitting patients with alcohol dependence and severe depression.^33^ Screening and brief intervention with family support was also super-efficacious versus screening and brief intervention alone, in relation to functioning among individuals with schizophrenia and problematic substance use.^33^ An Indian study highlighted the potential of screening and brief interventions to reduce the frequency of heavy drinking and alcohol risk scores.^31^

Home-based and community-based rehabilitation programmes, lasting 5–6 weeks, included psychoeducation, monthly visits, counselling and stress management. The findings were positive for reducing khat use; however, there was no evidence of effectiveness in reducing problematic alcohol use.^32,34^ A randomised controlled trial found no evidence of the effectiveness of community-based rehabilitation on alcohol use disorder (adjusted odds ratio 0.82, 95% CI 0.24–2.74; *P* = 0.74) at 12 months.^32^ Another study on a 6-week intensive home-based treatment programme demonstrated a reduction in khat use among out-patients.^29^

## Discussion

This scoping review aimed to provide an overview of current evidence on psychosocial interventions for individuals with severe MHCs and substance use in LMICs. We identified a limited number of primary studies focusing on intervention adaptation or testing in this population, with only four out of 13 studies being randomised controlled trials. These studies spanned eight LMICs, and their primary outcomes were varied, including substance use abstinence, treatment engagement and retention, reduction in psychiatric symptoms, functioning and suicidal behaviours.

The studies targeted a variety of substances, including tobacco, opioids, alcohol, khat and other drugs, using interventions such as early screening for dual conditions, tobacco cessation programmes, psychological interventions and psychosocial rehabilitation. However, there was no sufficient evidence on implementation processes, barriers or facilitators.

Some psychosocial interventions appear to be feasible and acceptable in LMICs among people with severe MHCs and SUDs.^40^ Tobacco cessation programmes, screening and brief intervention with family support, and home-based programmes have demonstrated positive outcomes in reducing the use of tobacco, alcohol and khat, respectively.^31,34,38^ However, the heterogeneity in outcomes, study designs and target populations made it difficult to draw concrete conclusions regarding the optimal type, mode, dosage and characteristics of psychosocial interventions for individuals with severe MHCs and co-occurring SUDs.

The feasibility and acceptability of the promising tobacco cessation programmes were largely limited to psychosocial care units. Various forms of CBT, such as brief CBT and mindfulness-based CBT, also demonstrated feasibility and acceptability in reducing depressive symptoms, addressing alcohol dependence and preventing substance relapse in settings like Iran and Thailand. Despite these successes, such interventions often required significant resources, including more trained healthcare providers, limiting their applicability in very low-resource settings.^28,33^

The existing evidence was mostly limited to tobacco and alcohol. However, psychostimulants like khat, which were more prevalent in some low-resource contexts such as East Africa, pose additional challenges for individuals with severe MHCs.^29^ Odenwald et al highlighted that although community-based interventions for psychosis were both feasible and acceptable in Somalia, challenges such as limited local capacity and comorbid khat use persisted. Therefore, adapting suitable interventions for people with severe MHCs and comorbid SUDs to low-resource settings remains critical.

There is an urgent need to promote guidelines for the screening and treatment of people with severe MHC and SUDs to achieve better outcomes, as concurrent disorders are often underdiagnosed, undertreated and more complex to manage.^41^ However, the current review found only one guideline from the Brazilian Association of Studies on Alcohol and Other Drugs (ABEAD) for the diagnosis and treatment of psychiatric comorbidity with alcohol and other substance dependence, which clearly described the scope and purpose of the guideline.^39^ Additionally, there was a study on the adaptation of an app to screen for dual disorders, which shows promise as a useful screening tool in residential addiction treatment centres.^37^ Previous global reviews identified 23 other guidelines, all of which were from HICs.^41^ The World Health Organization’s mental health Gap Action Programme Intervention Guide (mhGAP-IG 3.0) does not include specific interventions for the prevention and management of comorbidity between SUDs and severe MHCs. However, it recognises the detection of tobacco, alcohol and substance use among people visiting health facilities, and has potential for adaptation for people with severe MHCs.^42^ Universal screening for substance use in individuals with severe MHCs appears ethically and scientifically justified. However, it should be preceded by the development and evaluation of effective interventions. Therefore, addressing both severe MHCs and SUDs concurrently should be a priority.

This review also underscores inequities in healthcare provision for individuals with severe MHCs and SUDs.^43^ For individuals with severe MHCs and SUDs, even minimal substance use can have far-reaching consequences.^17–20,44^ Although not considered in this review, comorbidities like HIV/AIDS, hypertension, diabetes and cardiovascular diseases are often prevalent in this population,^21^ which underlines the need for a person-centred approach. Poor health outcomes in these populations can be attributed to barriers such as limited access to quality healthcare and routine screening, stigma from healthcare providers and a lack of knowledge about comorbid conditions. In some cases, substance use or physical health issues are overlooked or misinterpreted in individuals with severe MHCs, further complicating their treatment.^45,46^

The current study identified group therapy, CBT, brief interventions and MBRP as feasible psychosocial interventions in LMICs, in addition to psychosocial rehabilitation. In contrast, HICs have developed and tested a broader range of well-adapted psychosocial interventions for individuals with severe MHCs and SUDs.^22^ However, a Cochrane review indicated no clear superiority in efficacy among various psychosocial interventions – including CBT, motivational interviewing, case management, skills training and contingency management – either relative to each other or compared with treatment as usual.^22^ Although many psychotherapeutic approaches incorporate elements of motivational interviewing,^47^ evidence does not support its superior efficacy over other methods.^22^ Some interventions, such as assertive community treatment and token economy approaches, require substantial resources and may not be practical in LMICs settings. In contrast, low-resource interventions, such as motivational interviewing, psychoeducation and peer support, appear more promising. Additionally, many randomised controlled trials targeting severe MHCs exclude individuals with co-occurring substance use, thereby limiting the generalisability of their findings to dual diagnosis.^48^

We also found that cultural use of certain substances, such as khat, which is not widely recognised as an SUD, may be overlooked in studies of dual diagnosis.^48^ Khat is a stimulant with amphetamine-like properties,^49^ commonly used in East Africa and among diaspora communities in Europe.^50,51^ Its use has been significantly associated with elevated rates of psychotic symptoms. At least one psychotic symptom was reported by 16.8% of khat users.^50,52^ Psychosis has been particularly linked to heavy or prolonged khat use and may also exacerbate pre-existing MHCs.^52–54^ Khat use disorder has also been recognised as a valid diagnostic entity.^55^ Khat use disorder meets the core diagnostic criteria for stimulant use disorder as outlined in the DSM-5. These include impaired control over use, significant social and occupational harm, and characteristic withdrawal symptoms such as depressed mood, irritability, fatigue, lack of motivation, hypersomnia and increased appetite.^48,56^

The methodological quality of the studies included in this review was a concern. Many studies employed non-randomised, uncontrolled experimental designs, which come with high risk of bias.^57^ Some of the randomised controlled trials were underpowered to detect changes in the outcomes.^40^ Although better-powered studies may reveal significant differences, it might still be important to consider the clinical significance of the existing findings as they stand. This further highlights the need for more rigorous research on dual diagnosis in LMICs. In contexts like Ethiopia, where this review was conducted, designing and implementing psychosocial interventions presents challenges, including limited human and material resources. However, contextual adaptation of these interventions remains feasible.^58^

### Limitations of the scoping review

Because of the diversity of study designs, we were unable to conduct systematic risk-of-bias appraisal of the included studies. The wide variation in reported outcomes may have posed challenges in drawing strong conclusions across the evidence base.

In summary, our review has shown the limited efforts to design and test psychosocial interventions for individuals with severe MHCs and SUDs in LMICs. Unlike HICs, where evidence-based interventions exist, LMICs have not yet developed or tested effective solutions for this population. We strongly advocate for the design and testing of feasible, acceptable and effective interventions, such as dual screening programmes and integrated psychosocial interventions, to address both severe MHCs and substance use when they co-occur.

## Supporting information

Mihretu et al. supplementary material Mihretu et al. supplementary material

## Data Availability

All of the relevant data are included in this manuscript.

## Supplementary material

To view supplementary material for this article, please visit https://doi.org/10.1192/bjo.2025.10840
