# Supplementary material for: Psychosocial interventions for people with comorbid substance use disorders in people with severe mental health conditions in low- and middle-income countries: scoping review
Source: BJPsych Open. 2025 Sep 22;11(5):e222. doi: 10.1192/bjo.2025.10840 (PMC12458094; doi:10.1192/bjo.2025.10840)
Supplement: Mihretu et al. supplementary material [file S2056472425108405sup001.docx]

supplementary file 1**: Search terms**

((khat[Title/Abstract] OR alcohol[Title/Abstract] OR stimulant[Title/Abstract] OR cannabis[Title/Abstract] OR substance*[Title/Abstract] OR "substance use"[Title/Abstract] OR "problematic substance use"[Title/Abstract] OR substance use[MeSH Major Topic]) OR substance use disorders[MeSH Subheading] OR "severe mental"[Title/Abstract])

AND

("Major depression"[Title/Abstract] OR "psychotic disorders"[Title/Abstract] OR "dual diagnosis"[Title/Abstract] OR co-occurring[Title/Abstract] OR co-morbid[Title/Abstract] OR Schizophrenia[Title/Abstract] OR "bipolar disorder*"[Title/Abstract] OR "mood disorders"[Title/Abstract] OR "severe mental illness"[Title/Abstract] OR "mental illness"[Title/Abstract] OR "psychiatric disorders"[Title/Abstract] OR "Mental Disease"[Title/Abstract] OR Schizophrenia/[Title/Abstract] OR Psychosis/[Title/Abstract] OR "Psychotic Disorder"[Title/Abstract] OR "Affective Disorder"[Title/Abstract] OR "Severe Depression"[Title/Abstract] OR "Mental Disorder"[Title/Abstract] OR "Severe Mental"[Title/Abstract] OR Psychiatric[Title/Abstract] OR "Psychosocial Disabil*"[Title/Abstract])

AND

(intervention[Title/Abstract] OR trial[Title/Abstract] OR multicent*[Title/Abstract] OR study[Title/Abstract] OR evaluation[Title/Abstract] OR service[Title/Abstract] OR components[Title/Abstract] OR implementation[Title/Abstract] OR model[Title/Abstract] OR "pilot study"[Title/Abstract] OR feasibility[Title/Abstract] OR qualitative[Title/Abstract] OR implementation[Title/Abstract] OR pilot[Title/Abstract] OR "evaluation study"[Title/Abstract] OR "validation study"[Title/Abstract] OR care[Title/Abstract] OR multi-sectoral[Title/Abstract] OR strateg*[Title/Abstract] OR training[Title/Abstract] OR "clinical trial"[Title/Abstract] OR program*[Title/Abstract] OR treat*[Title/Abstract] OR "pilot study"[Title/Abstract] OR (Intervention[Title/Abstract] OR "psycho-social intervention"[Title/Abstract] OR psychotherapy[Title/Abstract] OR treatment[Title/Abstract] OR model[Title/Abstract] OR framework[Title/Abstract] OR therapy[Title/Abstract] OR "motivational interview"[Title/Abstract] OR "cognitive behavioral therapy"[Title/Abstract] OR "family therapy"[Title/Abstract] OR "psychoeducation"[Title/Abstract] OR training[Title/Abstract]))

AND

((low[Title/Abstract] AND middle-income countr*[Title/Abstract]) OR lmic[Title/Abstract] OR lamic[Title/Abstract] OR Afghanistan[Title/Abstract] OR Albania[Title/Abstract] OR Algeria[Title/Abstract] OR Angola[Title/Abstract] OR Antigua[Title/Abstract] OR Barbuda[Title/Abstract] OR Argentina[Title/Abstract] OR Armenia[Title/Abstract] OR Armenian[Title/Abstract] OR Aruba[Title/Abstract] OR Azerbaijan[Title/Abstract] OR Bangladesh[Title/Abstract] OR Benin[Title/Abstract] OR Belarus[Title/Abstract] OR Belize[Title/Abstract] OR Bhutan[Title/Abstract] OR Bolivia[Title/Abstract] OR Bosnia[Title/Abstract] OR Herzegovina[Title/Abstract] OR Botswana[Title/Abstract] OR Brazil[Title/Abstract] OR Bulgaria[Title/Abstract] OR Burkina Faso[Title/Abstract] OR Burundi[Title/Abstract] OR Cambodia[Title/Abstract] OR Cameroon[Title/Abstract] OR Cape Verde[Title/Abstract] OR Central African Republic[Title/Abstract] OR Chad[Title/Abstract] OR Chile[Title/Abstract] OR China[Title/Abstract] OR Colombia[Title/Abstract] OR Comoros[Title/Abstract] OR Congo[Title/Abstract] OR Costa Rica[Title/Abstract] OR Cote d Ivoire[Title/Abstract] OR Croatia[Title/Abstract] OR Cuba[Title/Abstract] OR Cyprus[Title/Abstract] OR Czech Republic[Title/Abstract] OR Djibouti[Title/Abstract] OR Dominica[Title/Abstract] OR Dominican Republic[Title/Abstract] OR East Timor[Title/Abstract] OR Ecuador[Title/Abstract] OR Egypt[Title/Abstract] OR El Salvador[Title/Abstract] OR Eritrea[Title/Abstract] OR Estonia[Title/Abstract] OR Ethiopia[Title/Abstract] OR Fiji[Title/Abstract] OR Gabon[Title/Abstract] OR Gambia[Title/Abstract] OR Georgia[Title/Abstract] OR Ghana[Title/Abstract] OR Grenada[Title/Abstract] OR Guatemala[Title/Abstract] OR Guinea[Title/Abstract] OR Guyana[Title/Abstract] OR Haiti[Title/Abstract] OR Honduras[Title/Abstract] OR India[Title/Abstract] OR Maldives[Title/Abstract] OR Indonesia[Title/Abstract] OR Iran[Title/Abstract] OR Iraq[Title/Abstract] OR Jamaica[Title/Abstract] OR Jordan[Title/Abstract] OR Kazakhstan[Title/Abstract] OR Kenya[Title/Abstract] OR Kiribati[Title/Abstract] OR Korea[Title/Abstract] OR Kosovo[Title/Abstract] OR Kyrgyzstan[Title/Abstract] OR Lao PDR[Title/Abstract] OR Latvia[Title/Abstract] OR Lebanon[Title/Abstract] OR Lesotho[Title/Abstract] OR Liberia[Title/Abstract] OR Lithuania[Title/Abstract] OR Macedonia[Title/Abstract] OR Madagascar[Title/Abstract] OR Malaysia[Title/Abstract] OR Malawi[Title/Abstract] OR Mali[Title/Abstract] OR Marshall Islands[Title/Abstract] OR Mauritania[Title/Abstract] OR Mauritius[Title/Abstract] OR Mexico[Title/Abstract] OR Micronesia[Title/Abstract] OR Moldova[Title/Abstract] OR Mongolia[Title/Abstract] OR Montenegro[Title/Abstract] OR Morocco[Title/Abstract] OR Mozambique[Title/Abstract] OR Myanmar[Title/Abstract] OR Namibia[Title/Abstract] OR Nepal[Title/Abstract] OR Netherlands Antilles[Title/Abstract] OR New Caledonia[Title/Abstract] OR Nicaragua[Title/Abstract] OR Niger[Title/Abstract] OR Nigeria[Title/Abstract] OR Oman[Title/Abstract] OR Pakistan[Title/Abstract] OR Palau[Title/Abstract] OR Panama[Title/Abstract] OR Paraguay[Title/Abstract] OR Peru[Title/Abstract] OR Philippines[Title/Abstract] OR Poland[Title/Abstract] OR Portugal[Title/Abstract] OR Qatar[Title/Abstract] OR Romania[Title/Abstract] OR Russia[Title/Abstract] OR Rwanda[Title/Abstract] OR Saint Kitts[Title/Abstract] OR Saint Lucia[Title/Abstract] OR Saint Vincent[Title/Abstract] OR Samoa[Title/Abstract] OR Sao Tome[Title/Abstract] OR Senegal[Title/Abstract] OR Serbia[Title/Abstract] OR Seychelles[Title/Abstract] OR Sierra Leone[Title/Abstract] OR Slovakia[Title/Abstract] OR Slovenia[Title/Abstract] OR Solomon Islands[Title/Abstract] OR Somalia[Title/Abstract] OR South Africa[Title/Abstract] OR South Sudan[Title/Abstract] OR Sri Lanka[Title/Abstract] OR Sudan[Title/Abstract] OR Suriname[Title/Abstract] OR Swaziland[Title/Abstract] OR Syria[Title/Abstract] OR Tajikistan[Title/Abstract] OR Tanzania[Title/Abstract] OR Thailand[Title/Abstract] OR Timor-Leste[Title/Abstract] OR Togo[Title/Abstract] OR Tonga[Title/Abstract] OR Trinidad[Title/Abstract] OR Tobago[Title/Abstract] OR Tunisia[Title/Abstract] OR Turkey[Title/Abstract] OR Turkmenistan[Title/Abstract] OR Tuvalu[Title/Abstract] OR Uganda[Title/Abstract] OR Ukraine[Title/Abstract] OR United Arab Emirates[Title/Abstract] OR United Kingdom[Title/Abstract] OR Uruguay[Title/Abstract] OR Uzbekistan[Title/Abstract] OR Vanuatu[Title/Abstract] OR Venezuela or Vietnam[Title/Abstract] OR Yemen[Title/Abstract] OR Zambia OR Zimbabwe[Title/Abstract])
